# Metastatic gallbladder carcinoma presenting as an ovarian mass

**DOI:** 10.4322/acr.2021.248

**Published:** 2021-04-06

**Authors:** Nidhin Rehman, Harish Sadashiva, Manoj Gopal Madakshira, Deep Kumar Raman

**Affiliations:** 1 Command Hospital, Department of Laboratory Medicine, Lucknow, India; 2 Command Hospital, Department of Oncosurgery, Lucknow, India

**Keywords:** carcinoma, gallbladder, immunohistochemistry, ovary

## Abstract

Metastatic gallbladder carcinoma to the ovaries is occasional but a recognized entity. It can mimic, clinical and morphologically, a primary ovarian tumor, challenging the diagnosis. We present the case of a patient with a lump in the hypogastrium extending into the right iliac fossa and was found to have abdominopelvic cystic lesion with enhancing solid components and multiple sub-centimetric and ill-defined abdominal lymph nodes. Also, subpleural and parenchymal nodules in the lungs were present. She subsequently underwent a laparotomy. Cholecystectomy was also done due to pre-existing symptomatic biliary lithiasis. The histologic report described the ovarian involvement as metastases from a gallbladder carcinoma. The presentation of ovarian metastases can challenge the diagnosis. Hence, careful evaluation of the digestive tract and judicious use of immunohistochemistry should be considered in patients presenting with ovarian masses.

## INTRODUCTION

The ovary is a frequent site of metastases from a wide variety of malignant neoplasms, mostly from the gastrointestinal tract. The gallbladder and bile ducts are rare sources of these metastases. Primary gallbladder cancer metastasizing to the ovary is infrequent and reported to be a meager 6%, and only a few cases have been reported.^1^ Many of these cases were initially misdiagnosed as a primary ovarian tumor. In these cases, the lack of awareness or limited information could cause an incorrect diagnosis.^2^ As gallbladder carcinoma also has a mucinous histology subtype, it becomes a diagnostic challenge to differentiate the primary ovarian cancer from the secondary when synchronous tumors are diagnosed. Furthermore, the similar non-specific clinical symptoms, signs, imaging findings, and tumor markers preclude the clinician from an accurate diagnosis.^3^ It is important to differentiate the two malignancies, as the management and prognosis are different. We report a case in which the patient presented with a lump abdomen. The patient was initially managed as a case of primary ovarian tumors. However, the gallbladder carcinoma identified incidentally during the histopathological examination after the exploratory laparotomy was the primary carcinoma, and the ovary was the metastatic focus.

## CASE REPORT

**Figure 1:**
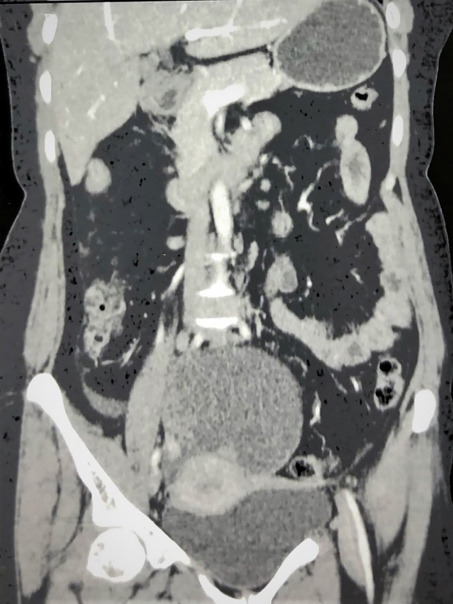
Magnetic resonance imaging (T1 weighted) shows a solid cystic ovarian mass with internal septations.

A 49-year-old female presented with complaints of right iliac fossa pain and lump abdomen for one year. There was no history of weight loss, loss of appetite, or early satiety. On examination, she was afebrile and well hydrated with stable vitals. There was no pallor, icterus, lymphadenopathy, or pedal edema. The thyroid and breast examination were normal. However, the abdominopelvic examination revealed a 10x8 cm lump in hypogastrium extending into the right iliac fossa with restricted mobility. The hematological and biochemical tests were normal. The serum tumor markers recorded values of: CA125 58.63 U/ml (reference value RV <46U/ml), AFP 4 ng/ml (RV: 10-20 ng/ml), β-HCG 2.12 mIU/ml (RV <5mIU/ml), and CA-19.9 17.2 IU/μl (RV: <37IU/ml). The pelvic ultrasonography revealed a heterogeneous cystic lesion with septations and solid components in the right adnexal region of 13.4 x 12 x 12cm. The abdominal contrast-enhanced computed tomography (CT) and Magnetic Resonance Imaging (MRI) revealed a well-defined abdominopelvic cystic lesion measuring 13x12x10cm with enhancing solid components of ovarian origin (Figure 1), and multiple subcentimetric and ill-defined subpleural and pulmonary nodules in the right upper lobe, right middle lobe and left upper lobe. The patient subsequently underwent a diagnostic laparoscopy. The intraoperative findings revealed a large adnexal mass, solid and cystic with minimal ascites and small sub-centimetric omental nodules. An omental biopsy was sent for histopathological examination, which showed fibrofatty tissue infiltrated by malignant cells seen as irregular glands eliciting a desmoplastic stromal response. In view of the above findings, optimal primary cytoreduction was feasible.

**Figure 2:**
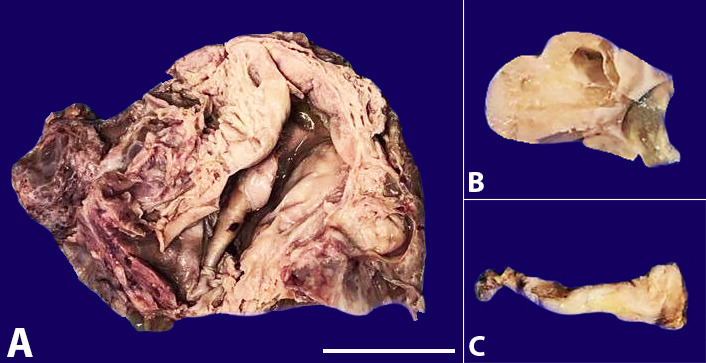
**A –** The cut surface of the right and left ovary shows a solid-cystic lesion with areas of necrosis (Scale bar: 3 cm); **B –** left ovary; **C –** thickened gallbladder wall.

Hence, she underwent staging laparotomy - Total abdominal hysterectomy with bilateral salpingo-oophorectomy along with bilateral pelvic as well as paraaortic lymphadenectomy and omentectomy, appendectomy, and cholecystectomy. In addition to the previous findings, intra-operative findings showed multiple pelvic lymph nodes, ascites, and thickened gallbladder. A frozen section was sent intra-operative from the ovarian mass indicated a borderline malignant tumor. On gross examination, the right ovary measured 13 x 10 x 05 cm and left ovary measured 06 x 03 x 03 cm. Both ovaries had a solid-cystic cut surface (Figure 2). The gallbladder measured 05 cm in length with an irregularly thickened wall measuring 1.5- 2.5 cm (Figure 2).

The maximum wall thickness was seen in the fundus with the puckering of the corresponding serosa. The mucosa was irregular.

**Figure 3:**
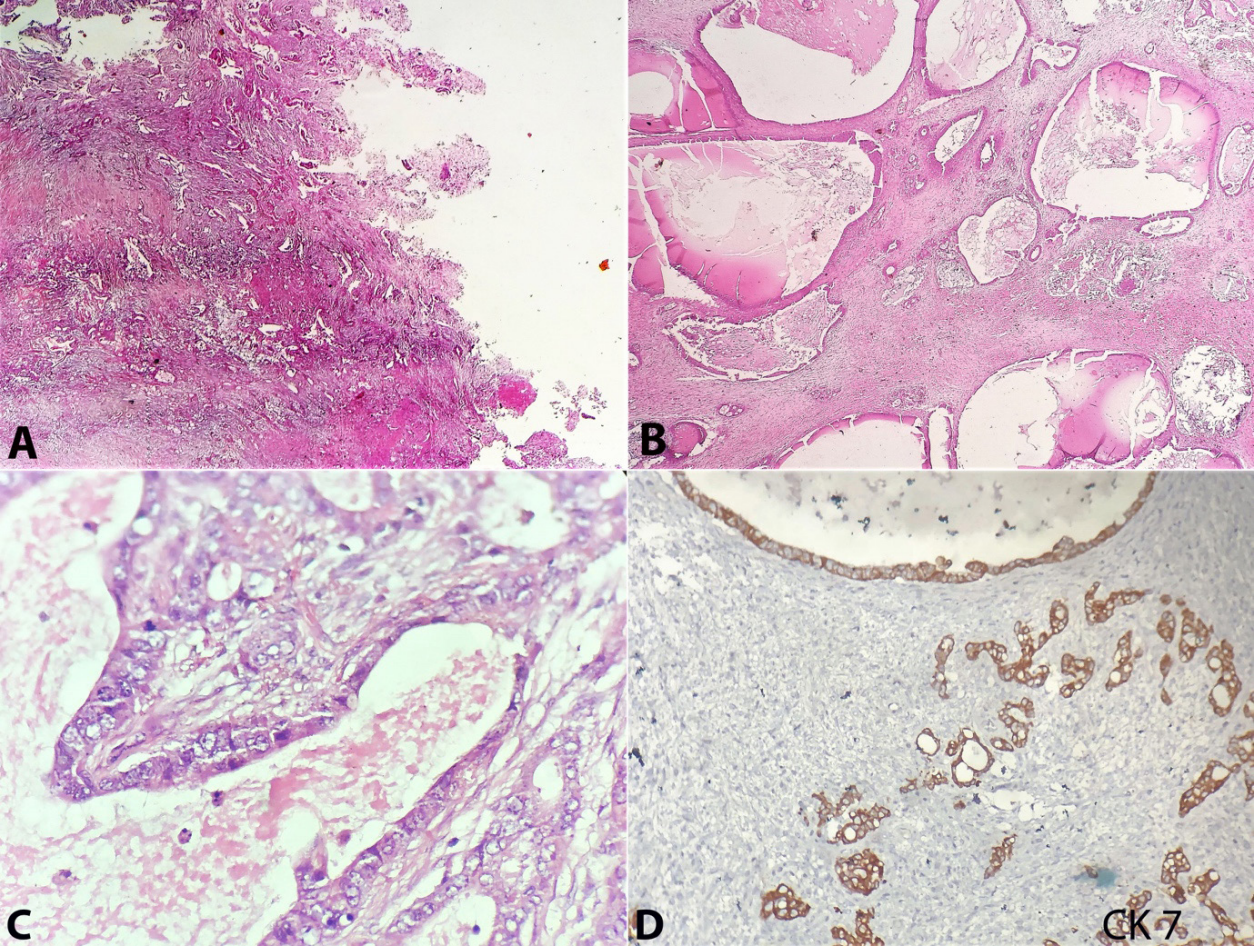
**A –** Photomicrograph of the gallbladder shows infiltrating irregular glands eliciting a desmoplastic response (H&E, 40x); **B, C** and **D –** Photomicrograph of right ovarian mass B - shows cystically dilated infiltrative glands (H&E, 40x); (C) shows the glands being lined by cuboidal to columnar cells having large vesicular nuclei, prominent nuclei and moderate amphophilic cytoplasm (H&E, 40x); (D) Cytokeratin 7 (40x magnification).

The histopathological examination from both ovaries showed an infiltrative tumor arranged in varying sized glands, many of which were cystically dilated, enclosing a pale mucinous material (Figure 3B). These glands were lined by cuboidal to columnar cells having basally placed large, pleomorphic nuclei with open to clumped chromatin, prominent nucleoli, and moderate pale eosinophilic to mucin filled cytoplasm (Figure 3C). The sections from the gallbladder showed a dysplastic mucosa along with underlying infiltrative glands, which are lined by cuboidal to columnar cells having similar morphology to those seen in both ovaries (Figure 3A). Perineural invasion was noted, and the tumor was seen to infiltrate the muscle to breach the serosa. Immunohistochemistry showed the tumor cells in the ovary as well as the in the gallbladder to be of the same phenotype, being positive for CK7 (Clone OV-TL, Pathnsitu, Ready to use) (intense, cytoplasmic), CK19 (Clone EP-73, Pathnsitu, Ready to use) (intense, cytoplasmic) and CDX2 (Clone EP-25, Pathnsitu, Ready to use) (focal, nuclear); while being negative for WT1 (Clone EP-122, Pathnsitu, Ready to use), ER (Clone EP1, Pathnsitu, Ready to use), PR (Clone EP2, Pathnsitu, Ready to use) and CK20 (Clone EP-23, Pathnsitu, Ready to use) (Figure 3 D, Figure 44B). P53 (Clone BP-53-12, Pathnsitu, Ready to use) showed mutant expression (50% cytoplasmic blush).

**Figure 4:**
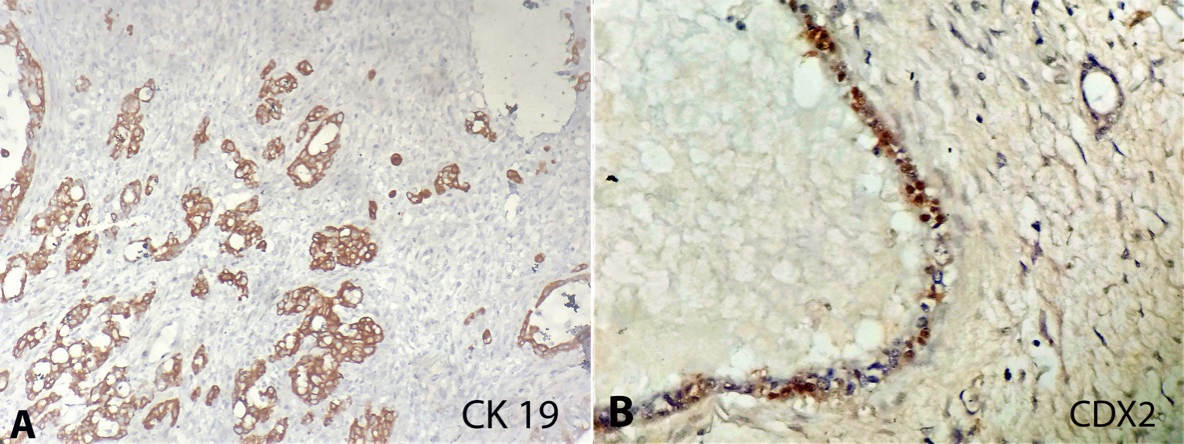
Photomicrographs of the ovary tumor. **A –** tumor cells positive reaction for cytokeratin 19 (40x); **B –** CDX2 shows nuclear staining in tumor cells (400x).

A final diagnosis of gallbladder carcinoma, Stage IVB (pT3NxM1) was made, considering only the para-aortic lymph nodes. The post-operative period was uneventful, and she was started on a gemcitabine-based palliative chemotherapy regimen.

## DISCUSSION

Metastatic neoplasms involving the ovary are sources of numerous problems in the differential diagnosis for the pathologist and have been the subject of much interest.^4^ Diverse issues have been emphasized, including but not limited to the remarkable mimicry of primary ovarian mucinous neoplasia in some cases. The morphology of metastatic intestinal adenocarcinoma, dating in large part from the seminal observations of Lash and Hart;^5^ and the delineation of a wider spectrum of the morphology of the Krukenberg tumor of the ovary than had previously been known for the most part.^6^ Additional patterns that were nonspecific have also complicated the morphology. However, there was also the small to medium-sized glandular pattern characteristic of many primary tumors of the biliary and pancreatic region.

The frequency with which the pathologist is confronted with a metastatic tumor of the gallbladder or extrahepatic bile duct origin varies worldwide because of the difference in the incidence of primary cancer. The clinical presentation of many tumors mimics that of primary ovarian neoplasia. The characteristics of gallbladder carcinomas with metastasis to the ovary on imaging may be similar to cholecystitis or cholelithiasis without obvious signs of polypoidal mass.^7^ Most patients present with nonspecific abdominal or pelvic symptoms (pain, distension, or mass), and a few, occasionally, present with jaundice. The operative findings can frequently note abnormalities in and around the primary site that will raise the suspicion of nonovarian origin. The surface involvement and a multinodular growth pattern are common, both very characteristic of metastasis.^6^ However, in our case, there was no evident surface involvement, and the gallbladder was removed only because the patient had a history of chronic cholecystitis. Morphologic heterogeneity from area to area within an individual slide is also very typical of metastasis.

Metastatic mucinous tumors can be very similar to the primary ovarian mucinous tumors, grossly. Our case showed the right ovary having multiple mucin-filled cystic areas along with many areas of necrosis. The solid areas were also mostly necrosed with evidence of hemorrhage. Grossly, the gallbladder did not show any mass, but its wall was diffusely thickened. Occasionally, these tumors pose major diagnostic problems microscopically, particularly when there is a bland-appearing epithelium or an adenofibroma or cystadenoma-like pattern. The presence of these bland and borderline-like areas could mislead one into concluding that the carcinoma arose out of a background primary ovarian neoplasm. Not infrequently, well-differentiated glands and cysts dispersed in prominent fibrous stroma strikingly mimicked ovarian adenofibroma, and similar well-differentiated cysts in a less conspicuous stroma mimicked a cystadenoma.^2^ Similar misinterpretation of such an area as a pre-existing ovarian lesion could lead one to wrongly conclude that the entire process was primary in the ovary. Awareness of this phenomenon and attention to the clinical findings, gross features, and other histologic indicators of metastatic disease, as noted previously, should enable one to avoid this diagnostic pitfall. However, in a limited sample, such as the intraoperative frozen section, this can be a particularly treacherous area.

In our case, the histopathological examination from both ovaries showed an infiltrative tumor arranged in varying sized glands, many of which were cystically dilated, enclosing pale mucinous material suggestive of a mucinous cystadenocarcinoma. However, the surprise came when the gallbladder sections showed a dysplastic mucosal lining along with underlying infiltrative glands having similar morphology as that seen in both the ovaries. In lieu of a high index of suspicion of a gallbladder carcinoma, the immunohistochemistry was done to differentiate between the two synchronous tumors. Though CK7/CK20 immune profile is extensively studied for differentiating colorectal cancer from mucinous ovarian carcinoma, data on their role in gallbladder carcinoma are limited. Our IHC showed the tumor cells in the ovary as well as the gallbladder to be of the same phenotype, being positive for CK7 (intense, cytoplasmic), CK19 (intense, cytoplasmic), and CDX2 (focal, nuclear); while being negative for WT1, ER, PR, and CK20. P53 showed mutant expression (50% cytoplasmic blush). This immune profile of CK7+/CK20-/CK19+/p53+ is typical of gallbladder carcinomas (GBC). In addition, GBCs have also known to express CEA, CA19-9, S100p, and MUC1.^8^ Primary mucinous ovarian tumors (PMOT) in contrast are usually unilateral, large, multicystic masses having a smooth surface, seldom showing infiltrative histology with nuclear expression of PAX8 and dual positivity for CK7 and CK20.^9^

The importance of differentiating primary gallbladder cancer from ovarian cancer is because of the different management. The 5-year survival rate of primary gallbladder cancer is abysmal (5%), whereas that of stage IV ovarian is 19%, and stage III ovarian cancer is (40-50%).^3^ Unlike primary ovarian cancers, the role of surgical management in gallbladder cancer is limited to early-stage disease. The use of either radiotherapy or chemotherapy provides little improvement in survival for patients with advanced primary gallbladder cancers. Moreover, chemotherapy regimens for both are different.^10^^,^^11^ For unresectable gallbladder carcinomas, the preferred regimen is Gemcitabine based while Platinum-based regimens are preferred for advanced Ovarian carcinomas.^10^^-^^12^

## CONCLUSION

Occult gallbladder carcinoma presenting initially as a metastatic tumor to the ovary has not been widely studied. These tumors can closely mimic primary ovarian mucinous tumors. In spite of extensive cross-sectional radiological imaging and serum markers, an accurate diagnosis may not be possible preoperatively. Pathologists should always maintain a high index of suspicion, and adequate sampling should be done of bilateral ovarian masses. In all bilateral mucinous tumors, the outer surface should be sifted through for the presence of tiny deposits. Knowing the extent to which gallbladder metastases may mimic a primary ovarian tumor, differentiating histological features, and appropriate use of immunohistochemistry will help make the correct diagnosis and provide direction for further patient management.
